# Anticipated nursing care: findings from a qualitative study

**DOI:** 10.1186/s12912-020-00486-y

**Published:** 2020-10-06

**Authors:** Michela Bottega, Alvisa Palese

**Affiliations:** 1 Department of Biomedicine and Prevention, University of Rome “Tor Vergata”, Via Montpellier, 1 - 00133, Rome, Italy; 2 Department of Medical Sciences, University of Udine, Viale Ungheria, 20, 33100 Udine, Italy

**Keywords:** Anticipated nursing care, In-depth interview, Missed nursing care, Nursing activities, Nursing interventions, Qualitative study

## Abstract

**Background:**

Contrary to Missed Nursing Care, some anecdotal data and sparse evidence has documented the tendency of nurses to anticipate some nursing interventions. However, no study has been conducted to date with the purpose of understanding this phenomenon and its underlying mechanisms and consequences. The aim of this study was to describe the phenomenon of delivering anticipated nursing care, its antecedents and consequences as perceived by nurses.

**Method:**

A descriptive qualitative study. The Consolidated Criteria for Reporting Qualitative Research guidelines were followed. A purposeful sample of 17 clinical nurses and nurse managers working in three Italian hospitals were interviewed in depth in 2019. The audio-recorded interviews were verbatim transcribed and thematically analysed.

**Results:**

‘Anticipated Nursing Care’ is delivered significantly earlier than when expected by nurses in their care plan, by patients, by caregivers and by other members of the team. Medication administration, mobilisation of patients, hygiene care, changes of dressing, vital parameter monitoring, blood sampling and administrative activities were reported as interventions delivered before rather than when expected. Clinically stable patients have been reported to be at risk of receiving anticipated nursing care. Individual values and attitudes, group attitudes of being always ready for the *“unexpected”*, implicit group norms to *“leave the patients and the unit in order”*, high workloads, intertwined activities and work processes inside the units, have been reported as reasons for Anticipated Nursing Care. Effects of this phenomenon have been reported at the patients’ and at the nurses’ level.

**Conclusion:**

Anticipated Nursing Care occurs when nurses perform interventions earlier than expected according to an implicit or explicit decision and not as a consequence of a request. The phenomenon requires future studies to detect its diffusion and to accumulate evidence. Its presence in daily practice, if confirmed, suggests that Missed Nursing Care studies should also consider the combined effect of these two phenomena as, on one hand, there may be the tendency to postpone and, on the other hand, the tendency to anticipate interventions.

## Summary box

What does this paper contribute to the wider global clinical community?

- Anticipated Nursing Care is present in clinical practice and occurs when nurses perform some interventions earlier than expected according to an implicit or explicit decision and not as a consequence of a request (e.g., from patients).
- Medication administration, mobilisation and hygiene care, dressing changes, vital parameter monitoring, blood sampling and documentation are all interventions at risk of being anticipated.
- Nurses individual values and attitudes, nursing staff group attitudes, implicit norms, high workloads, intertwined activities and work processes inside the units, trigger anticipating nursing care.
- Consequences of Anticipated Nursing Care are both negative and positive, at the patient and at the nurses’ level.

## Background

In recent years, concepts such as Missed Nursing Care [1, 2], Care Left Undone [3, 4], Unfinished Nursing Care [5, 6], Rationing of Nursing Care [7, 8], and Compromised Nursing Care [9], hereafter Missed Nursing Care (MNC), have been studied by several researchers and countries becoming an international field of interest.

Conceptually, MNC is defined as any aspect of required patient care that is omitted (either in part or in whole) or delayed [1, 2]. MNC has been associated with the lack of resources and unbalanced skill-mix (e.g. [9–13]), implicit and explicit team rules [14], nurses’ decision-making processes [8], increased number of patients (e.g., increased admissions [15];) and communication issues inside of the team (e.g. [2]). Amongst all the documented factors leading to MNC, time scarcity has been suggested to guide the decision of which intervention is a priority [6] among potentially competitive nursing interventions. As a consequence, some interventions have a greater risk of being postponed or omitted [14, 15], leading to negative outcomes [12]. For its relevance, MNC has been the focus for several researchers (e.g. [9–13, 15]) aimed at understanding predictors and interventions that are capable of minimising its occurrence. However, in its research history the phenomenon of MNC was first identified by Kalisch [1] in a qualitative study where 25 focus groups were performed with nurses, nursing assistants, and unit secretaries in two hospitals. In this study, it was emerged the first operational definition of MNC, the elements of interventions missed on a regularly basis (e.g., ambulation, turning), and seven themes expressing the possible reasons for missing care emerged. Based on this work, Kalisch and colleagues further delineated the concept of MNC by performing a concept analysis [1].

Contrary to MNC, some anecdotal data and sparse evidence have documented the tendency of nurses to anticipate - instead of postponing or omitting - some nursing interventions. Concrete examples are night nurses waking patients cared for early in the morning; or nurses providing hygiene care very early in the morning, and preparing patients for the night by doing these activities before breakfast and just after dinner, respectively. There is also evidence of nurses conducting procedures in advance such as preparing medications by leaving them on bedside tables, starting the medication administration before the provisional time [16]. This anecdotal data, which seem to reflect rituals and routines that tend to be repeated over time [17, 18], are accompanied also by some empirical evidence. This is the case of leaving a medication at the patient’s bedside in advance [19]. Moreover, despite recommendations regarding that the administration of antibiotic prophylaxis in non-clean and implant surgery should take place within 60 min prior to incision, empirical evidence has reported that prophylaxis is administered more than 120 min prior to incision [20]. Furthermore, there are reports of peripheral cannulas being inserted in the Emergency Department in just admitted patients to ensure venous access; however, around 18.2% of them are later not used [21] suggesting therefore that these might have been positioned prematurely, without assessing the need for it.

On one side, the anecdotal and empirical examples available seem to highlight a phenomenon strictly consequent of a process of prioritisation of nursing care [22] where nurses attribute a rank order, importance or time towards competing alternatives, thus starting with some (e.g., administrating antibiotic prophylaxis) and ending with those documented at risk of being delayed or omitted. Along this line, it can be argued that researchers to date have focused their efforts on the delayed or omitted interventions (e.g. [5, 6, 22]): therefore, it is time to consider the entire process where some interventions are also provided in an anticipated manner to ensure that all are delivered. Moreover, on the other hand, nurses can decide to anticipate some nursing care according to their experience [23] or time-management issues, that have been also reported as anecdotal in nursing care [24]. Specifically, time management in nursing practice has been reported as an externally-defined set of practices [25] at need of more empirical research and analysis [25]: in fact, studies available are a few and documenting nurses who compensate for lack of time by developing strategies in an attempt to complete their work. However, no specific studies are documented to date by valuing the tacit knowledge of nurses [26] as to why they try to anticipate nursing actions (e.g., waking or mobilising patients early) by allocating these actions in a different time than that expected.

Understanding this phenomenon and its underlying mechanisms can improve the knowledge available regarding the process of prioritisation of nurses on how they actively negotiate care delivery in complex situations and where workloads and staffing are challenging [27]. Moreover, as with delaying or missing interventions, anticipating care can also have consequences on patients and the nursing care flow: therefore, discovering the possible consequences can increase the understanding of the impact of nursing care on patients. Contributing to advancing the knowledge in the field was the general aim of this study.

## Methods

### Aims

To describe the practice of nursing care delivered in an anticipated manner as compared to when expected, its antecedents and consequences according to the experience of nurses.

### Design

A descriptive-qualitative study design [28] was conducted. The study design was selected according to its capacity to locate the merging findings within the existing anecdotal and sparse empirical knowledge regarding the phenomenon of interest [29]. Therefore, the study was based on a deductive – inductive strategy, in that the ‘Anticipated Nursing Care’ concept as reported in empirical and anecdotical data [16–21, 30] was shared with participants, and then elicited data about their experience of the phenomenon. The Consolidated Criteria for Reporting Qualitative Research (COREQ [31];) has been followed in reporting the methods and the findings as outlined in Supplementary Table 1.

### Participants

Nurses were the target population. Specifically, clinical nurses (=Registered Nurses, RNs) and nurse managers (NMs) of three hospitals located in northern Italy were involved. Then, a purposeful sample of participants [32] that reflected a maximal variation of experiences [33] and capable of offering an in-depth description of the phenomenon of interest [34] was approached. The clinical nurses and the nurse managers were included based on the following criteria: (a) they were working in general medical and surgical acute hospital settings, (b) had at least almost 1 year of work experience, thus ensuring a complete familiarisation with the context and the nursing care [35], and (c) gave their written informed consent to participate in the study. Those clinical nurses and nurse managers who did not satisfy the inclusion criteria as well as those who had reported a previous working or personal relationship with the interviewer [31], were all excluded.

The clinical nurses were identified by the nurse managers of the unit involved while the nurse managers were identified by the nurse director of the hospital. The progressive inclusion was ended at the data saturation [36, 37] that was assessed by two researchers in an independent fashion when no new data was elicited.

### Data collection

Data collection was performed in 2019 through a semi-structured interview [38] as reported in Table 1. An interview guide was developed with open-ended questions with the intent of generating a free and in-depth reflection regarding the topic under study and by considering the study performed by Kalisch [39] who first developed the concept of MNC. The interview guide was firstly piloted among three clinical nurses with the purpose of establishing its feasibility, clarity, and ability to stimulate reflections on the phenomenon of interest. No changes were required after the pilot phase.

**Table 1:** Semi-structured interview question guide

Areas under investigation	
General data Gender, age, nursing education, years of nursing experience; current role (RN, NM); unit (medical or surgical).	
Questions 1_ Might you describe your direct experience or indirect experience of witnessing other colleagues, of nursing care interventions conducted in an anticipated manner rather than at their expected time?	
2_ Might you describe the nursing interventions that you experienced or witnessed to be anticipated most frequently in your daily nursing care?	
3_ Might you share your perceptions regarding why these interventions were anticipated? Who takes the initiative to anticipate nursing interventions? For which patients? Might you report the conditions when this phenomenon occurred in your experience (e.g. shifts …).	
4_ What effects did you observe in patients exposed to nursing interventions that were delivered early?	
5_Please feel free to share other elements of this phenomenon.	

*NM* Nurse Manager, *RN* Registered Nurse

Then, potential participants were first approached by email with a brief explanation of the purpose of the study and the request to participate; then, they were contacted personally by the Principal Investigator (MB) with a phone call to arrange the face-to-face interview appointment.

The interviews were performed in a place decided by participants, in a calm and undisturbed environment, when they were not involved in the clinical care of patients. Each interview was also audio-recorded [38], after having obtained the written informed consent.

### Ethical considerations

The study protocol was approved by the Belluno and Treviso (Italy) Provincial Ethic Committee on the 27th May 2019 (n. 678/CE Marca). All participants gave their voluntary written informed consent prior to study participation. Aimed at ensuring privacy and confidentiality, each participant interview was anonymised with a progressive number (e.g., first Registered Nurse interviewed, RN1) while quotes extracted were also anonymised (e.g., hospital units).

### Data analysis

In line with the study design, researchers were interested in examining the nurses’ experiences by analysing their narratives as manifested forms of beliefs and constructs [40]. Thus, two researchers openly shared their preconceptions [41] regarding the phenomenon and its meaning for clinical nurses and nurse managers with the intent of preventing biases in data analysis.

A thematic analysis [41] was conducted, aimed at describing and qualifying the phenomenon of interest. Firstly, the audio-recorded interviews were verbatim transcribed (MB); then, these were read and re-read by two researchers (see authors) to obtain a global view and to familiarise with the data. Therefore in the first stage of analysis, initial codes were generated [42] manually, by underlying the transcripts and then reporting these in the left-hand margin as interesting or significant segments. In the second stage of analysis, the emerged codes were collapsed into potential themes and each was defined by developing a clear definition (Supplementary Table 2). In the third stage, the themes were also connected to each other by generating a conceptual map. In producing the report, selected quotes from the interviews were included in an anonymous fashion, without differentiating those emerged from RNs and those from NMs. Two researchers performed the entire process in an independent manner; in each step, they shared and agreed on the findings.

### Rigor and trustworthiness

During the interviews, no feedback, negative or positive reinforcements were offered to participants to ensure freedom in sharing their experience [38]. Moreover, for the purpose of ensuring credibility, (a) transcribed interviews were reported to participants for comments and corrections, making the member check it with them [38], and (b) a prolonged engagement in the research process [43] was ensured by researchers, who were involved for more than 1 year.

To ensure dependability of consistency in data collection procedures [44] different strategies were adopted: (a) the interview guide was the same for participants, (b) one researcher (MB, PhD student, female, at the time of the study she was working in the Nursing Office of a large Health Care Trust, North of Italy) was involved in the data collection; and (c) two researchers conducted the data analysis (MB and AP, PhD, female, Associate Professor in Nursing Science, the Dean of the Bachelor of Nursing programme, in the North of Italy). None were involved in the care of patients.

Moreover, to ensure confirmability of the findings [44] (a) direct quotes were extracted (e.g., RN1), (b) an audit trail was provided (Supplementary Table 2), and (c) several meetings were performed to discuss emerging codes and themes until a consensus was reached [45].

## Results

### Participants

Fifteen clinical nurses and four nurse managers were approached; two nurses refused to participate for personal reasons, specifically, they did not have time for interviews after their shifts. As reported in Table 2, the average age of participants was 39.4 years (Standard Deviation [SD] 9.2); the majority were female (16/17) with > 13 years of experience (SD 9.7) on average as a nurse and > 7 years on average (SD 7.1) in the current medical or surgical unit. Three nurses were working part-time at the time of the study. The interview duration was, on average, 34 min and ranged from 29 to 50 min.

**Table 2:** Participants

Interview number^a^	Age Group	Education	Current Role	Experience as a nurse (Years)	Experience the unit (Years)	Unit	Working time	Interview duration (Minutes)
1	51–60	Nursing Diploma; Post-Diploma Education in Management	NM	21	6	Surgery	FT	43
2	41–50	Bachelor of Nursing Degree; Post-Graduate Education in Management	NM	9	6	Surgery	FT	30
3	41–50	Bachelor of Nursing Degree	RN	27	14	Surgery	FT	39
4	31–50	Bachelor of Nursing Degree	RN	10	10	Surgery	PT 24 h/week	37
5	51–60	Nursing Diploma; Post-Diploma Education in Management	NM	33	17	Surgery	FT	36
6	41–50	Bachelor of Nursing Degree; Post-Graduate Education in Management	NM	2	2	Medical	FT	28
7	21–30	Bachelor of Nursing Degree	RN	5	2	Medical	FT	44
8	21–30	Bachelor of Nursing Degree	RN	5	1.5	Surgery	FT	30
9	41–50	Bachelor of Nursing Degree	RN	23	6	Surgery	PT 27 h/week	29
10	21–30	Bachelor of Nursing Degree	RN	6	0.5	Medical	FT	30
11	31–40	Bachelor of Nursing Degree	RN	7	2	Surgery	FT	25
12	21–30	Bachelor of Nursing Degree	RN	6	2	Medical	FT	43
13	31–40	Bachelor of Nursing Diploma; Post-Graduate Advanced Education	RN	14	1.5	Medical	FT	28
14	41–50	Bachelor of Nursing Diploma	RN	25	23	Medical	PT 24 h/week	36
15	41–50	Bachelor of Nursing Diploma	RN	10	10	Medical	FT	50
16	21–30	Bachelor of Nursing Diploma	RN	4.5	3	Medical	FT	29
17	41–50	Bachelor of Nursing Diploma	RN	25	20	Medical	FT	29

*F* Female, *FT* Full-Time, *M* Male, *NM* Nurse Manager, *PT* Part-Time, *RN* Registered Nurse

### Anticipated nursing care: the concept

According to the nurses’ experiences, ‘Anticipated Nursing Care’ is the intervention(s) delivered significantly earlier than the time expected by nurses in their care plan, by patients, by relatives and caregivers, and by other members of the team. Therefore, according to their experience, the practice of ‘Anticipated Nursing Care’ does not reflect the process of “prepping” for tasks before they are scheduled to occur. It reflects interventions delivered in an anticipated manner or prematurely on a regular basis rather than occasionally or in critical situations. The decision to anticipate nursing care is taken by nurses and not as a result of an explicit or implicit request of anticipation, for example by patients (e.g., the emerging new need), relatives (an intervention when they are still in the hospital) or physicians (e.g., giving a medication in advance). Different words have been used to express this anticipation:

*“moving forward”* (RN2, RN3, RN7, RN9, RN10, RN12, RN14, RN15, RN16, RN17),

*“to anticipate”* (RN4, RN8, RN13, RN15),

*“playing in advance”* (RN9, RN10, RN11),

*“carrying on*” the activities (RN4, RN12, RN13),

*“gaining an advantage”* (RN1),

*“going on like a train”* (RN5),

*“taking more time to do better”* (RN6),

*“doing everything from start to finish”* (RN7).

Nurses have reported that stable and collaborating patients have an increased risk of receiving anticipated nursing care (RN1, RN4, RN5, RN7, RN8, RN10, RN11, RN15) as well as those who are visited by relatives on a regular basis (RN12).

### Anticipated nursing care: the interventions

Not all nursing interventions have been reported to be anticipated. The ones more at risk of being anticipated are medication administrations, patient’s mobilisation, hygiene, dressing changes, the vital parameter monitoring, the blood sampling and some administrative procedures.

Medication administration has been underlined by all participants as the most often anticipated intervention in three main aspects: (a) by diluting, in advance, medications that need to be administrated intravenously (RN1, RN6, RN12), (b) by leaving the pills in dosettes near the bedside table when the patient is stable, oriented and collaborating or with a planned discharge (RN3, RN4, RN5, RN6, RN9, RN14), and (c) by starting the administration of medications with the trolley > 60 min in advance (e.g., the medications expected at 4 pm are provided at 2 pm or 3 pm) to ensure that all patients receive the medications required (RN2, RN7, RN8, RN10, RN11, RN13, RN14).

In the context of early mobilisation after surgery or after an acute event with the aim of preventing post-surgical complications or pressure sores (RN2, RN3, RN5, RN11, RN12, RN14, RN15, RN17), nurses have reported performing these interventions in advance while, for example, they are in the patient room to perform another planned activity in order to optimise time by avoiding the need to come back to the same room later.

Morning hygiene, such as a complete shower or bed hygiene, has been reported to be anticipated in the early hours or at the end of the night (RN3, RN7, RN9, RN13, TN15, RN10); similar reports have emerged regarding bowel management (e.g., enema administration) (RN3, RN4, RN7, RN12, RN13). Along this line, preparing patients for nights has been reported as performed during the evening shifts (RN2, RN3, RN4, R15).

Nursing procedures have also been used to be anticipated in time: it has been reported that surgical sites (RN1, RN3) and devices or ostomies (RN4) that no longer need to be cared for have had their dressings changed at the end of the night shifts or early in the morning (RN5, RN9, RN15, RN17). Vital signs are monitored in advance (RN1, RN3, RN4, RN7, RN8, RN9) while blood samples planned for the mornings (8 am) have been documented to be performed by night shift nurses (RN1, RN2, RN3, RN7, RN8, RN10, RN11, RN12, RN16) before 7 am.

Furthermore, some administration procedures have been anticipated, such as completing the documentation of clinical records regarding a stable patient before the end of the shift (RN1, RN3, RN7, RN8, RN10, RN12, RN14, RN15), preparing blood samples and diagnostic examinations request forms before their actual prescription (RN1, RN2, RN5, RN6, RN7, RN8, RN9, RN10, RN11, RN11, RN12; RN14, RN15, RN16), or preparing documentation regarding the patient’s transfer to another unit (e.g., rehabilitation RN2, RN4, RN5). In addition, the provision of medications or other material supports required by the unit are requested in advance before the resources are actually finished (RN2, RN4, RN10, RN16).

### Anticipated nursing care: the antecedents

**Fig. 1:**
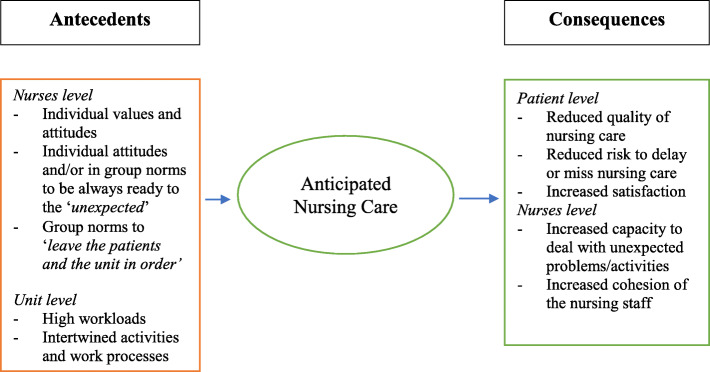
Anticipated Nursing Care: Antecedents and Consequences

As reported in Fig. 1, the following elements emerged as reasons for Anticipated Nursing Care: (a) individual values and attitudes, (b) individual and/or group attitude to always be ready for the “*unexpected*”, (c) implicit group norm to “*leave the patients and the unit in order*”, (d) high workloads, and (e) intertwined activities and work processes.

Personal values and attitudes have been reported as having a role in the decision to anticipate the nursing care: they have been reported as an expression of a personality trait (RN1, RN3) of nurses who need to keep the interventions under control in the fear of missing some of them (RN4). Therefore, with the purpose of preventing any missed care, these are immediately done. On the other hand, the desire to be a well-performing nurse (RN1, RN8, RN13, RN14, RN15) has been reported as triggering Anticipated Nursing Care.

Individual attitudes to anticipate the care in order to be ready for the “*unexpected*” in the case of a sudden increased volume of patients (e.g., more admissions or discharges, complicated patients in need of care; RN2, RN4, RN5, RN10, RN11, RN12, RN15) have emerged. These individual attitudes have been reported to be transformed into norms in well-established groups with an internal cohesion and low turnover of staff. In these conditions, all possible interventions are anticipated for stable patients (RN1, RN3, RN4, RN6, R10, RN11, RN17), thus leaving time free for those with an unpredictable and increased need of nursing care during the shifts. In this light, expert nurses have been reported to decide to anticipate nursing care when they perceive that the situation of the unit may worsen (e.g., increased risk of understaffing) or there are signs of variations in the complexity of patients or in the workloads of the unit (RN1, RN4, RN6, RN9, RN11, RN13, RN14, RN15, RN16, RN17).

Participants also reported unwritten nursing staff rules that require them to *“leave the patients and the unit in order”* for the next colleagues (RN5, RN6, RN9, RN11, RN13, RN16, RN17) by doing all activities before the shift ends (RN2, RN4, RN10, RN11, RN13, RN14, RN16, RN17) to prevent an unfavourable start for the next shift. In other words, all activities should be performed and no additional duties should be left to the following shift (RN1, RN2, RN4, RN6, RN7, RN10, RN12). This prevents the following colleagues from forgetting some interventions (RN1, RN13, RN17) and allows them to finish all activities within their own working shift (RN8, RN13, RN16, RN17) as a sort of *“sign of respect”* for the next colleagues (RN2).

The need to face high workloads and the attempt to optimise different intertwining activities and processes have been reported as influencing the decision of the nursing staff to anticipate interventions. The unfavourable nurse-to-patient-ratio and the attempt to guarantee the nursing care required to all patients have been reported as the main factors triggering Anticipated Nursing Care (RN6, RN7, RN8, RN9, RN10, RN11, RN12, RN13, RN14, RN15, RN16). As a consequence, for example, device medications and enemas are often conducted during night shifts to optimise the time necessary to “*touch the patient*” and “*stay in his/her room*” only once (RN1, RN4, RN7, RN9, RN11, RN12). The administration of medications early in the morning has been reported to allow them to be taken by patients when the nursing aides help them with breakfast (RN6, RN7, RN8, RN9, RN10, RN11, RN12) or to allow the medical records with prescriptions to be available before the beginning of a physician’s visit to a patient (RN1, RN15).

Therefore, some interventions have been reported to be anticipated to coordinate nurses and nursing aides (RN3) and nurses with physicians by completing nursing care before being interrupted by the medical staff or in order to complete all planned activities (e.g., hygiene) when the physicians arrive on the units (RN1, RN2, RN3, RN4, RN5, RN6, RN7, RN8, RN9, RN10, RN11, RN13, RN14, RN15). With regards to the extra units’ interconnections, some interventions have been reported to be anticipated to align the workflow of the unit with other units, such as the laboratory, emergency, radiology and physiotherapy services (RN3, RN6, RN11), who are involved in the patients’ clinical path.

No differences in the occurrence of Anticipated Nursing Care have been reported by participants during the week compared to weekend shifts or across shifts (RN1, RN2, RN3, RN4, RN8, RN16).

### Anticipated nursing care: the consequences

As reported in Fig. 1, at the patient level, nurses reported that the quality of nursing care can be threatened by anticipating nursing care (RN1, RN7, RN8, RN9, RN10, RN11, RN13, RN15). However, they also underlined that patients are satisfied to receive care in advance as a sign of nurses’ attention and proactivity (RN1, RN3, RN7). Furthermore, anticipating activities for some patients, such as dispensing medications in advance, has been reported as allowing nurses to reduce the risk of delaying or missing nursing care (RN2, RN3, RN4, RN5, RN6, RN7, RN9, RN12, RN15).

At the nurses’ level, anticipating nursing care allows them to be able to manage any critical issues within the unit such as unexpected problems and activities (RN2, RN3, RN9, RN7, RN11). Moreover, at the group level, Anticipated Nursing Care has been reported positively, increasing the cohesion and the sense of respect, particularly if the following shift has new staff that may be slower in completing the nursing care required by patients (RN2, RN4, RN8, RN12). By anticipating activities, nurses can end some nursing interventions (e.g., patient hygiene, medication administration) before the medical staff’s planned activities (e.g., bedside visit), favouring multi-professional work (RN1, RN2, RN3, RN4, RN5, RN6, RN7, RN8, RN9, RN10, RN11, RN13, RN14, RN15).

## Discussion

This study was aimed merging the phenomenon of delivering Anticipated Nursing Care, its antecedents and consequences as perceived by nurses. To date only anecdotical or sparse empirical evidence of the phenomenon have been reported in the literature (e.g., [18–23]). Therefore, we have mirrored the work of the pioneer Professor Kalisch who merged the opposite concept, namely the Missed Nursing Care, with a study performed with a qualitative descriptive methodology which was the basis later of a concept analysis. Involving the nurses and giving to their experience voice with a qualitative study based upon individual interviews, have been considered important considering that nurses might feel guilty, powerless to correct the situation or fearful regarding unsatisfactory nursing practice [1, 2].

According to the findings, Anticipated Nursing Care implies not just an intervention performed before another as a consequence of the order attributed to interventions in the process of prioritisation or time-management [21, 24] aimed at managing time scarcity [46] and where a sort of chain is designed, and thus some interventions are implemented first and others later. By anticipating nursing care, nurses perform some interventions earlier than expected as implicitly or explicitly deliberated by themselves and not as a consequence of an external request (e.g., from patients). The fact that interventions are prematurely delivered mainly to stable patients reinforces that this phenomenon is not a consequence of a simple prioritisation where problems are categorised into those that require immediate actions and those that can be left because they are not urgent [22, 47].

Nurses have used different words to indicate the Anticipated Nursing Care phenomenon as “*moving forward*”, “*anticipating*” and “*playing in advance*”, suggesting that they try to adjust the flow of activities by doing something prematurely. Therefore, alongside MNC, where interventions are postponed and then missed [39], an opposite phenomenon seems to exists. Interestingly, the majority of anticipated interventions have the same risk of being delayed and/or postponed. According to studies available [48], the most frequent interventions to be delayed or missed have been reported as, in order, turning and positioning patients, performing oral hygiene and bathing, administering medications and reporting full data in the documentation. These have also emerged as anticipated in our study. On the other hand, interventions reported at lower risk of being missed or delayed such as vital sign monitoring, glucose controlling and skin care [48] have also been reported to be anticipated in our study. Furthermore, for patients that are more at risk of not receiving an intervention in time, while anticipated nursing care has emerged as more often delivered to stable patients and those who have a family member at their bedside, MNC has been reported to affect certain patients such as those that are older or with certain non-urgent diseases (e.g., dementia [49]). Less severely ill patients receive lower priority [50]. This seems to confirm that in the field of MNC and Anticipated Nursing Care, a potential source of discrimination among patients in need of nursing care might exist [51].

Two main reasons for Anticipated Nursing Care emerged, the first at the nurse level (individual or group) and the second at the unit level, all mirroring those already reported for the MNC phenomenon [1, 2, 7, 8]. Among them, individual values, such as professional nursing principles serving as a framework for standards, professional practice and evaluation [50] and individual or group attitudes, such as the relatively constant manner of thinking, feeling and behaving [52], that can all become norms as cognitive standards about the acceptability of a behaviour [53] have emerged.

Individual values and attitudes to be performant and to prevent MNC have emerged among the causes of Anticipated Nursing Care [8] suggesting that allocating early an intervention is mainly decided at the individual level by nurses according to their clinical reasoning and decision-making processes. Kalisch and colleagues, in their eminent works [1, 2] have identified the role of habits and values. On one hand, nurses can be driven individually and then as a group according to the moral obligation [50] to perform all activities planned, leading them to anticipate all that is possible and feasible. It is also possible that moral obligation is learned since nursing students’ education [54] through the example of expert nurses who anticipate the nursing care. On the other hand, it may be possible that the implicit norms of the Italian nursing practice of being recognised by colleagues and recognising themselves as ‘*a good nurse’* is to be capable of performing all planned activities on time. In this context, alongside the moral obligation, there is also the need to be recognised as a ‘*good nurse’*. This can explain why, in the Italian context, despite the poor nurse-to-patient ratios (around 1:13 or more as documented in medical units, [27]), the MNC occurrence has been reported as similar to that documented in other countries where a more favourable nurse-to-patient ratio has been reported [13, 15, 48, 55–57]. Moreover, with the increased number of patients, Italian nurses have been reported to reduce the MNC occurrence [27]: this paradox can be explained by the effect of some compensatory mechanisms relying on anticipating nursing care when faced with increased workloads.

All these possible explanations of Anticipated Nursing Care are supported by another reason, namely “*leaving the patients and the unit in order*”: in other words, the tendency of nurses to only find themselves at ‘*peace’* after having performed all their tasks. This seems to be not only an indicator of good performance as an individual nurse but also a sign of team respect and commitment [58] to the incoming nurses responsible for the next shift. They will be exposed to the same work pressure where preventing any additional tasks from being postponed until the next shift, or better anticipating something, thus leaving time for unexpected needs, has become a group norm.

Being ready for the unexpected has been reported as a reason for Anticipated Nursing Care; by anticipating tasks, nurses attempt to reduce the risk of omitting or delaying interventions and allow the staff to feel safer when facing unexpected situations [10, 14]. Hospital care is characterised by increased acuity of patients, as well as increased volume of patients admitted from the emergency departments, suggesting that nurses shape their decision-making processes on the nursing work flows around this. Moreover, intertwined activities and work processes of other members of the team have been reported as a cause of Anticipated Nursing Care while communication issues have already been documented as a cause [12]. Findings emerged in our study seem to reflect a work process design where different teams and units impose their time to nurses that are under pressure to consequently adapt their work process to this pressure and not to the patients’ needs. Nurses have already been reported to be at risk of prioritising routine tasks over patients’ needs [59] and this might be determined by the need to coordinate the nursing process with other complex work processes of other teams.

Patients who received care in advance have been reported by nurses to be more satisfied and at less risk of being exposed to MNC. Patient dissatisfaction with nursing care has been associated with MNC [48] while receiving care on time or early can prompt the perception of being a high priority by nurses. Thus, differently to MNC, Anticipated Nursing Care has a positive effect at the patients’ level and at the nurses’ level, thus it is a phenomenon at risk of being perpetuated given its positive implications. However, nurses also recognised that by anticipating nursing care, the quality of care has an increased risk of being poor. In this line, disturbing sleep, as in the case of early waking, as well as threatening the daily rhythms of the patient (when having hygiene, when being mobilised, when going in to the bed), which are all part of the Fundamentals of Care [60, 61], have been reported.

### Limitations

The study has several limitations. Firstly, only units pertaining to a public hospital trust, in northern Italy were approached and involved. Variations across the country due to the federalisation of the healthcare system at the regional level [62] have already been reported, suggesting the need to further investigate the phenomenon in other contexts and also internationally. Secondly, we have involved clinical nurses and nurse managers: differently, nursing aides have been largely involved in studies measuring MNC (e.g., [14, 27, 63]). Nursing aides have different educational backgrounds, scope of practice and responsibilities towards patients and families [27]; however, their further involvement can provide a full picture of the phenomenon under study by reviewing other anticipated care aspects under their control. Thirdly, with regards to the antecedents, these have emerged from participants working in acute care contexts; therefore, future studies should investigate if the same causes are reported in other settings such as long-term and community care. Fourth, with regards to the methodology used, we have not performed a concept analysis which is required to establish the theoretical foundations of the Anticipated Nursing Care near to the experienced of nurses emerged in this study; the same concept ‘Anticipated Nursing Care’ has been established a priori, considering the words used in the daily practice by Italian nurses confirmed by also the quotes emerged in the study. However, defining the best term to be use require a concept analysis that it is strongly encouraged.

### Relevance to clinical practice

There is a high risk that Anticipated Nursing Care will be perpetuated due to its positive visible effects. Understanding values and attitudes at the individual and at the group level to design educational opportunities capable of improving the decision-making processes regarding how to manage time scarcity can be useful. At the education level, given that nurses’ norms can pressure students to replicate behaviour, strategies to ensure the time to perform the interventions required by patients on time as well as opportunities to reflect on the underlining reasons for both Anticipated and MNC are strongly suggested. Moreover, strategies aimed at redesigning work processes at the unit and hospital levels to prevent anticipated interventions that result from the forced alignment applied over the nursing care processes with other work processes, is strongly suggested.

Alongside policy level strategies aimed at ensuring appropriate care resources to prevent Missed and Anticipated Nursing Care, the phenomenon of anticipated care requires future studies at the international level to develop its diffusion and to accumulate evidence in the field. Its presence in daily practice, if confirmed, suggests that studies aimed at discovering the causes and the effects of MNC should also consider the combined effect of these two phenomena as, on the one hand, the tendency to postpone and, on the other hand, the tendency to anticipate interventions are in a constant conflict with respect to when activities (as for example that regarding the fundamental needs [64]) should be allocated.

## Conclusions

Anticipated Nursing Care is present in clinical practice and occurs when nurses perform some interventions earlier than expected according to an implicit or explicit decision and not as a consequence of a request (e.g., from patients). Those at risk of receiving Anticipated Nursing Care are stable patients. Interventions at risk of being anticipated are medication administration, mobilisation and hygiene care, dressing changes, vital parameter monitoring, blood sampling and documentation. Individual values and attitudes regarding who should be considered a good nurse, as well as values and attitudes that become norms, such as always being ready for the unexpected or the need to leave the patients and the unit in order, have been reported as reasons leading to the Anticipated Nursing Care at the nurses’ level. Moreover, high workloads and the need to adapt the process of care to the intertwined activities and work processes of other teams and/or units have been reported at the unit level. Both negative, such as the decreased quality of nursing care, and positive effects, such as the increased satisfaction and the reduction of MNC, have been reported at the patients’ level. Future studies are needed to get insights into the processes whereby nurses make decisions about practices that bring forward care.

## Supplementary information

**Additional file 1 Supplementary table** Analysis of the study conduction according to the COnsolidated criteria for REporting Qualitative research [32].

**Additional file 2 Supplementary Table 2** Coding tree: examples.

## Data Availability

The datasets used and/or analysed during the current study are available from the corresponding author on reasonable request.

## Abbreviations

COREQ: Consolidated Criteria for Reporting Qualitative Research
F: Female
FT: Full-Time
MNC: Missed Nursing Care
NA: Not Applicable
PT: Part-Time
NM: Nurse Manager
RN: Registered Nurse
SD: Standard deviation
