# Supplementary material for: Anticipated nursing care: findings from a qualitative study
Source: BMC Nurs. 2020 Oct 6;19:93. doi: 10.1186/s12912-020-00486-y (PMC7541304; doi:10.1186/s12912-020-00486-y)
Supplement: Supplementary file 1 — Additional file 1 Supplementary table Analysis of the study conduction according to the COnsolidated criteria for REporting Qualitative research [32]. [file 12912_2020_486_MOESM1_ESM.docx]

**Supplementary table** Analysis of the study conduction according to the COnsolidated criteria for REporting Qualitative research [32]

| **No. Item** | **Guide questions/description** | **Reported on Page No.** |
| --- | --- | --- |
| **Domain 1:** **Research team and reﬂexivity** | |  |
| **Personal Characteristics** | |  |
| 1. Interviewer/facilitator | Which author/s conducted the interview or focus group? | Methods, Rigor and trustworthiness, page 7-8 |
| 2. Credentials | What were the researcher’s credentials? E.g. PhD, MD | Methods, Rigor and trustworthiness, page 7-8 |
| 3. Occupation | What was their occupation at the time of the study? | Methods, Rigor and trustworthiness, page 7-8 |
| 4. Gender | Was the researcher male or female? | Methods, Rigor and trustworthiness, page 7-8 |
| 5. Experience and training | What experience or training did the researcher have? | Methods, Rigor and trustworthiness, page 7-8 |
| **Relationship with participants** | |  |
| 6. Relationship established | Was a relationship established prior to study commencement? | Methods, Data collection, page 6 |
| 7. Participant knowledge of the interviewer | What did the participants know about the researcher? | Methods, Data collection, page 6 |
| 8. Interviewer characteristics | What characteristics were reported about the interviewer or facilitator? | Methods, Data collection, page 6 |
| **Domain 2: Study design** | |  |
| **Theoretical framework** | |  |
| 9. Methodological orientation and Theory | What methodological orientation was stated to underpin the study? | Methods, Design, page 5-6 |
| **Participant selection** | |  |
| 10. Sampling | How were participants selected? | Methods, Participants, page 6 |
| 11. Method of approach | How were participants approached? | Methods, Data collection, page 6 |
| 12. Sample size | How many participants were in the study? | Results, Participants, page 6 |
| 13. Non-participation | How many people refused to participate and why? | Results, Participants, page 6 |
| **Setting** | |  |
| 14. Setting of data collection | Where was the data collected? e.g. home, clinic, workplace | Methods, Data collection, page 6 |
| 15. Presence of non-participants | Was anyone else present besides the participants and researchers? | Methods, Data collection, page 6 |
| 16. Description of sample | What are the important characteristics of the sample? | Results, Participants; Table 1 |
| **Data collection** | |  |
| 17. Interview guide | Were questions, prompts, guides provided by the authors? Was it pilot tested? | Methods, Data collection; Table 2 |
| 18. Repeat interviews | Were repeat interviews carried out? If yes, how many? | No |
| 19. Audio/visual recording | Did researchers use audio/visual recording to collect the data? | Methods, Data collection, page 6 |
| 20. Field notes | Were ﬁeld notes made during and/or after the interviews? | Methods, Data collection, page 6 |
| 21. Duration | What was the duration of the interviews or focus group? | Results, Participants, page 6 |
| 22. Data saturation | Was data saturation discussed? | Results, Participants, page 6 |
| 23. Transcripts returned | Were transcripts returned to participants for comment and/or correction? | Methods, Rigor and trustworthiness, page 7-8 |
| **Domain 3: Analysis and ﬁndings** | |  |
| **Data analysis** | |  |
| 24. Number of data coders | How many data coders coded the data? | Methods, Data analysis, page 7 |
| 25. Coding tree description | Did authors provide a description of the coding tree? | Supplementary Table 2. |
| 26. Derivation of themes | Were themes identiﬁed in advance or derived from the data? | Methods, Data analysis, page 7 |
| 27. Software | What software, if applicable, was used to manage the data? | N/A |
| 28. Participant checking | Did participants provide feedback on the ﬁndings? | No |
| **Reporting** | |  |
| 29. Quotations presented | Were participant quotations presented to illustrate the themes/ﬁndings? | Results, page 8-12 |
| 30. Data and ﬁndings consistent | Was there consistency between the data presented and the ﬁndings? | Methods, Rigor and trustworthiness, page 7-8 |
| 31. Clarity of major themes | Were major themes clearly presented in the findings? | Results, page 8-12 |
| 32. Clarity of minor themes | Is there a description of diverse cases or discussion of minor themes? | Results, page 8-12 |

*N/A* Not applicable

**Supplementary table 2** Coding tree: examples

|  | **Categories** | **Codes** | **Quotes, participant** |
| --- | --- | --- | --- |
| **The phenomenon** | **Anticipated Nursing Care** | Care delivered significantly early (prematurely) or before the time as expected | “Moving forward” (RN2, RN3, RN7, RN9, RN10, RN12, RN14, RN15, RN16, RN17)  “To anticipate” (RN4, RN8, RN13, RN15) |
| **The interventions** | **Medication** | (a) diluting medication in advance  (b) leaving the pills in dozers near the bedside table  (c) starting the administration of the medications with the trolley also > 60 minutes in advance | “The drugs dilution tends to be anticipated… yes especially the dilution” (RN1)  “For oral medications, the nurse prepares the pills, leave them on the bedside table...” (RN6)  “If I have to administer the medication at 8am because it is written at 8am, I try to start as soon as possible, which is still 7am. Therefore, given that I have 15 patient who expect the medications, some will have it at 7.30am, others will have it at 9.30 am” (RN10) |
| **The antecedents** | **Implicit group norms** | Implicit group norm to “*Leave the patients and the unit in order*” | “Not leaving something to do the colleagues of the next shift” (RN1) |

*RN* registered nurse
