# Supplementary material for: Anticipated nursing care: findings from a qualitative study
Source: BMC Nurs. 2020 Oct 6;19:93. doi: 10.1186/s12912-020-00486-y (PMC7541304; doi:10.1186/s12912-020-00486-y)
Supplement: Supplementary file 2 — Additional file 2 Supplementary Table 2 Coding tree: examples. [file 12912_2020_486_MOESM2_ESM.docx]

**Supplementary table 2** Coding tree: examples

|  | **Categories** | **Codes** | **Quotes, participant** |
| --- | --- | --- | --- |
| **The phenomenon** | **Anticipated Nursing Care** | Care delivered significantly early (prematurely) or before the time as expected | “Moving forward” (RN2, RN3, RN7, RN9, RN10, RN12, RN14, RN15, RN16, RN17)  “To anticipate” (RN4, RN8, RN13, RN15) |
| **The interventions** | **Medication** | (a) diluting medication in advance  (b) leaving the pills in dozers near the bedside table  (c) starting the administration of the medications with the trolley also > 60 minutes in advance | “The drugs dilution tends to be anticipated… yes especially the dilution” (RN1)  “For oral medications, the nurse prepares the pills, leave them on the bedside table...” (RN6)  “If I have to administer the medication at 8am because it is written at 8am, I try to start as soon as possible, which is still 7am. Therefore, given that I have 15 patient who expect the medications, some will have it at 7.30am, others will have it at 9.30 am” (RN10) |
| **The antecedents** | **Implicit group norms** | Implicit group norm to “*Leave the patients and the unit in order*” | “Not leaving something to do the colleagues of the next shift” (RN1) |

*RN* registered nurse
